# Semi-automatic FISH quantification on digital slides

**DOI:** 10.1186/1746-1596-8-S1-S21

**Published:** 2013-09-30

**Authors:** Gábor Kiszler, László Krecsák, Annamária Csizmadia, Tamás Micsik, Dániel Szabó, Viktor Jónás, Viktória Prémusz, Tibor Krenács, Béla Molnár

**Affiliations:** 1 Department of Image Analysis, 3DHISTECH Ltd., H-1121 Budapest, Konkoly-Thege Miklós u. 29-33, Hungary; 2 Ist Department of Pathology and Experimental Cancer Research, Semmelweis University, H-1085 Budapest, Üllöi u. 26, Hungary; 3 H-1063 Budapest, Podmaniczky u. 63, Hungary

## Background

HER2 is a transmembrane glycoprotein, a member of epidermal growth factor receptor family. It was documented that the amplification and the over expression of this gene plays an important role in the pathogenesis and in the progression of breast cancer [1]. Nowadays this is one of the most important biomarker and target for breast cancer therapy. 10-30% of invasive breast carcinomas are HER2 positive, the gene over expression occurring in invasive ductal adenocarcinomas and in invasive lobular carcinomas as well. Therefore the assessment of the HER2 receptor status of the formalin-fixed paraffin embedded cancer specimens has a key importance for specifying the appropriate therapy. For prognostic and predictive testing the immuncytochemical and FISH stains are routinely used in the clinical diagnostic. According to the guideline of the American Society of Clinical Oncology/College of American Pathologists [2] when the immunoquantification is not clear FISH stain should be apply to support the diagnosis. By using dual or multicolor probes, the chromosomal aberration and gene sequence modification can be detected in parallel.

Several methods are available for the evaluation of gene status in FISH stained samples, such as the analysis of the image histogram [3]. Netten et al. [4] used image processing tools to develop their own algorithm, which automatically detects the region of interest, counts the cell nuclei and finds the spots in each nucleus. Furthermore, there is an algorithm which quantifies 2D and 3D FISH images as well [5]. All of these methods work on images which were recorded by microscope. The conventional microscopic diagnosis process allows to make quantification on the recorded sub-regions of the sample. With the expansion of the digital pathology, we intended to develop a FISH quantification platform, which combines the innovative fluorescence whole slide scanning technology with image processing applications.

## Material and methods

### Samples and scoring

Two TMA samples with 20-19 representative HER2 cases were used in this study. Samples were selected from the archive of the 1st Department of Pathology and Experimental Cancer Research of the Semmelweis University, Budapest, Hungary. The survey was performed with the permission of the Institutional Review Board and Regional Ethics Committee of the Semmelweis University (RKEB) (permit no. 7/2006). Sample selection was based on the original IHC HER2 (c-erbB-2 / HER-2 / neu Ab-12, Thermo Fisher Scientific Inc. USA) scoring in order to use cases that cover all positivity ranges. Thus 24 HER2 positive and 16 HER2 negative cases were included in the TMAs. The formalin-fixed paraffin embedded specimens were stained using HER2 FISH pharmDx™ kit (K5331, Dako, Denmark). Scanned was performed using Pannoramic 250 FLASH (3DHISTECH Ltd., Hungary), that utilizes Plan-Apochromat 40x magnification, 0.95 numerical aperture objectives (Zeiss, Germany) and a PCO.edge 5.5 megapixel, scientific Complementary Metal-Oxide Semiconductor camera (Kelheim, Germany) for fluorescent image acquisition. One part of the image optimalization processes was integrated in the Pannoramic 250 FLASH scanner, which can set up the white balance and can make a shedding correction and a special fluorescent compensation as well. By applying the extended focus scanning method, the different recorded focus lanes (Z=5, step size=0,8 µm) were represented in one summarized virtual lane, which formed the basis of the image processing. The three fluorescent channels were recorded separately and represented the inputs of our FISH detection algorithm.

### Integrated development environment

We used Visual C++ for developing the image analysis software application. The IPP (Intel Performance Primitives) library was used for image processing.

### Test process

After the whole slide scanning, all TMA cores were scored by an expert pathologist, using Pannoramic Viewer 1.15.2 ver. software (3DHISTECH Ltd., Hungary). Maximum 40 cell nuclei were quantified, as detailed in the HER2 FISH pharmDx™ kit scoring guideline.

Comparisons were made between the semi-automated assessments, the scorings performed using the software platform and the original ICH scores.

As the data obtained show a non-normal distribution, non-parametric tests (Cohen’s kappa and Spearman rank-correlation) had to be applied, using the MedCalc for Windows v. 11.2.1.0 (MedCalc Software, Mariakerke, Belgium) software application. The strength of agreement was interpreted as proposed by Landis & Koch [7].

## Results and discussion

### Image analysis

**Figure 1:**
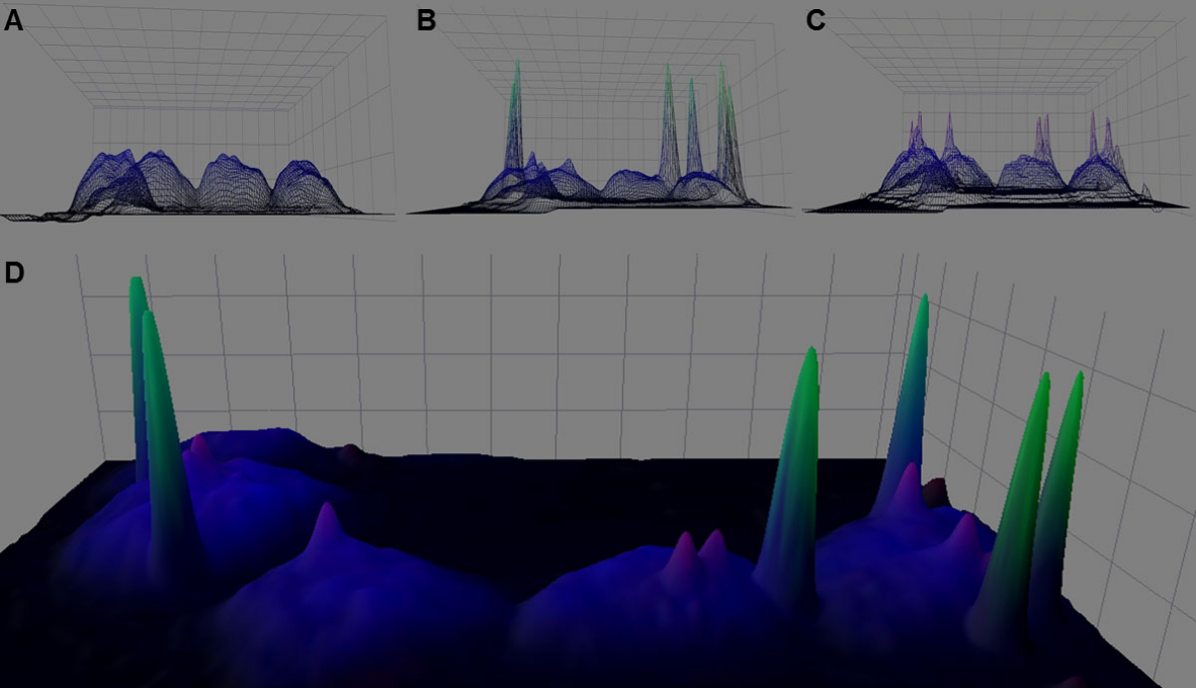
The results of the intensity analysis of the digital slide. The DAPI (A), FITC (B) and the rhodamine (C) channels after intensity amplifying. Signals are represented with different intensity in the summarized image (D), therefore an optimal threshold had to be defined in each channel.

First we analyzed the blue (DAPI) channel, which represented all cell nuclei. The intensity dynamics and the geometrical morphology of the epithelial cell nuclei were investigated (Figure1A). After a locally adaptive contrast enhancement the intensity profile of the nuclei followed smooth shape, where the most frequent pixel intensities were from the central chromatin of the cell nuclei. A specific slope graph could be defined at the border of the nuclei in the region of the marginal chromatin and the nuclear membrane. Using these findings, the intensity and the geometrical features of an ideal cell nucleus could be described. This “ideal nucleus” was used hereafter as a nuclei filter in the detection algorithm.

After extended focus recording, all FISH spots were represented in focus plane. Thus the geometrical and intensity parameters of the signals were similar in one channel (Figure 1B, 1C, 1D). This allowed us to apply a general spot detection algorithm for the whole slide. Our algorithm used a local intensity amplifying mechanism, followed by a threshold process, the latter dependent on the quality of the digital slide.

The actual spot numbers were identified for each of the detected cell nucleus and based on this the cell nuclei were classified using the usual Her2 classification rule. Three groups were defined; normal (red signal and green signal were represented equally in the cell nucleus), amplification (the ratio of the red signal and green signal was higher than one per nucleus) and artifact (others).

### Preliminary validation

A Microscopical Image Segmentation Profile (.misp file) was defined based on the morphological parameters of the nuclei (i.e. radius, area and circularity), the contrast and the intensity of the slide. This profile was used to make investigation on 39 TMA spots. The results of the measurement were saved into an Extensible Markup Language (XML) file and were compared with the results of the Her2 immunostain.

**Figure 2:**
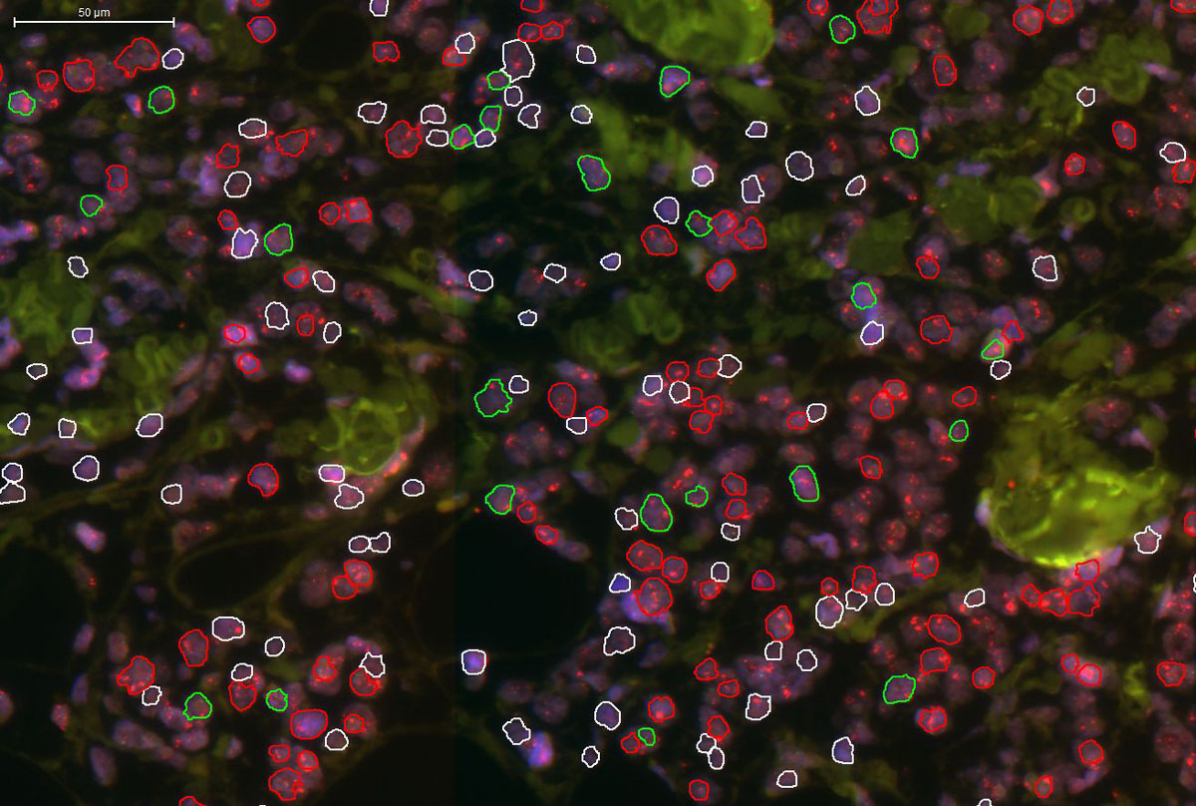
The images results after the cell nuclei quantification. Different outlines show the classified nuclei: red=amplification, green=normal, white=non classified, artifact. The cell nuclei were filtered based on their shape, thus only the rounded cell nuclei were classified.

Results of the TMA cores (Figure 2) could be used in the statistical analysis, in diverse extents. The number of nuclei identified and scored using the software platform within each TMA core higly exceeded (mean 279.84 cells, min. 26-max. 939 cells) the number of cells suitable for enumeration of borderline cases (i.e 40 cells) as defines in the FISH pharmDx™ kit scoring guideline. Therefore the ratio of normal and amplified cells has been standardized to 40 cells/TMA core prior to conducting the statistical analysis.

An almost perfect agreement has been found between the semi-automated scoring and the results provided by the software platform (Table 1.). Apposite to this result, both the semi-automated investigation and the automated scoring showed only substantial agreements with the initial ICH HER2 score.

**Table 1:** Agreement between the different scorings.

Variables	Cohen’s κ (95% CI)	Spearman’s rho (df, 95% CI, p)
Semi-automated scoring vs Software platform	0.911 (0.750-1.0)	0.992 (25, 0.981-0.996, <0.0001)
Semi-automated scoring vs Initial ICH score	0.606 (0.322-0.890)	0.659 (25, 0.357-0.836, =0.0003)
Software platform vs Initial ICH score	0.703 (0.498-0.909)	0.762 (39, 0.588-0.869, <0.0001)

## Conclusions

The basis of our algorithm was the description of the intensity and morphometric features of an “ideal nucleus”. This was used to identify cell nuclei with ideal diagnostic potential.

We tested our image analysis application under routine conditions. The selected TMA samples were produced by the histopathology labor and scored manually by an expert pathologist based on the IHC stains and the virtual FISH slide. The results were compared to the measurements of the software. The selection of a high number of cells for FISH scoring did not alter the final results, as showed by the almost perfect agreement found beetween the semi-automated scoring and the one provided using the software application. The use of automated application allow users to select and investigate any sample to a greater extent and with more precison as they would do by using the conventional, manual microscopic, assessment.

The FISH quantification is usually run according to the investigation of fluorescent samples. The quantification process is visually harmful and cumbersome because of the dark room and the bright mercury lump light illumination. The combination of the whole slide digitalization with image segmentation makes the microscopic investigations more accurate and reproducible. Moreover this technic make possible to detect and quantify high numbers of cell nuclei on the whole territory of the recorded samples and analyze fluorescent signals without bleaching.

## List of abbreviations

FISH: Fluorescens In Situ Hybridization; IHC: Immunohistochemistry; Her2: Human Epidermal growth factor Receptor 2; TMA: Tissue Micro Array; DAPI: 4',6-diamidino-2-phenylindole, a fluorescent cell nuclei stain; FITC: Fluorescein isothiocyanate, a fluorescent stain; XML: Extensible Markup Language

## Competing interests

Tamás Micsik MD and Tibor Krenács MD are working as a consultant for 3DHISTECH Ltd. for 5 years.

## Author contributions

GK: made digitization and wrote the majority of the manuscript

TM: pathologist, performed immunohistochemical evaluation,

AC: performed and evaluated FISH-reactions and analyzed the samples with FISHQuant

DS and VJ: optimized the and developed image segmentation

LK: performed the statistical analysis and wrote a part of the manuscript

TK: pathologist, worked as consultant during this work

BM: worked as consultant during this work, managed company affairs
